# COVID-19 screening in low resource settings using artificial intelligence for chest radiographs and point-of-care blood tests

**DOI:** 10.1038/s41598-023-46461-w

**Published:** 2023-11-11

**Authors:** Keelin Murphy, Josephine Muhairwe, Steven Schalekamp, Bram van Ginneken, Irene Ayakaka, Kamele Mashaete, Bulemba Katende, Alastair van Heerden, Shannon Bosman, Thandanani Madonsela, Lucia Gonzalez Fernandez, Aita Signorell, Moniek Bresser, Klaus Reither, Tracy R. Glass

**Affiliations:** 1 Radboud University Medical Center, 6525 GA Nijmegen, The Netherlands; 2 SolidarMed, Partnerships for Health, Maseru, Lesotho; 3 Centre for Community Based Research, Human Sciences Research Council, Pietermaritzburg, South Africa; 4 SAMRC/WITS Developmental Pathways for Health Research Unit, Department of Paediatrics, School of Clinical Medicine, Faculty of Health Sciences, University of the Witwatersrand, Johannesburg, Gauteng South Africa; 5 Department of Infectious Diseases and Hospital Epidemiology, University Hospital Basel, Basel, Switzerland; 6 SolidarMed, Partnerships for Health, Lucerne, Switzerland; 7 Swiss Tropical and Public Health Institute, Allschwil, Switzerland; 8 University of Basel, Basel, Switzerland

**Keywords:** Diagnosis, Computational science, Biomedical engineering

## Abstract

Artificial intelligence (AI) systems for detection of COVID-19 using chest X-Ray (CXR) imaging and point-of-care blood tests were applied to data from four low resource African settings. The performance of these systems to detect COVID-19 using various input data was analysed and compared with antigen-based rapid diagnostic tests. Participants were tested using the gold standard of RT-PCR test (nasopharyngeal swab) to determine whether they were infected with SARS-CoV-2. A total of 3737 (260 RT-PCR positive) participants were included. In our cohort, AI for CXR images was a poor predictor of COVID-19 (AUC = 0.60), since the majority of positive cases had mild symptoms and no visible pneumonia in the lungs. AI systems using differential white blood cell counts (WBC), or a combination of WBC and C-Reactive Protein (CRP) both achieved an AUC of 0.74 with a suggested optimal cut-off point at 83% sensitivity and 63% specificity. The antigen-RDT tests in this trial obtained 65% sensitivity at 98% specificity. This study is the first to validate AI tools for COVID-19 detection in an African setting. It demonstrates that screening for COVID-19 using AI with point-of-care blood tests is feasible and can operate at a higher sensitivity level than antigen testing.

## Introduction

As the SARS-CoV-2 (coronavirus) pandemic unfolded around the world in 2020, scientists and clinicians scrambled for tools to aid with diagnosing the associated respiratory illness, coronavirus disease 2019 (COVID-19). The gold standard tests—reverse transcription polymerase chain reaction (RT-PCR)—were in short supply worldwide and additionally took approximately 2 days to produce a result. Antigen-based tests were still being developed and were not widely available. At the same time, health services around the world experienced large numbers of people presenting with respiratory symptoms and urgently needed to determine which patients should be isolated and treated for COVID-19. A large body of scientific literature emerged at that time, detailing various routes through which COVID-19 could potentially be diagnosed, particularly using imaging or laboratory markers. These methods included analysis of chest imaging including computed tomography (CT) or X-Ray^1–6^ and investigation of blood markers^7–12^. There has additionally been a great deal of work on artificial intelligence (AI) methods to automatically diagnose COVID-19 using CT imaging^13–22^, chest X-Rays^23–34^ , audio samples^35,36^, or blood test results^37–41^. The chest X-Ray (CXR) is a low-cost image which can be acquired using portable equipment and operated by personnel with minimal training, making it a very useful diagnostic tool in resource-constrained settings. Many commercial products have been launched for the detection of tuberculosis (TB) on CXR^42^ in regions where TB is endemic and resources are limited. Furthermore, while many areas of the world do not have access to hospital laboratories, point-of-care (POC) blood testing can be achieved at low cost and with immediate results. Both CXR and POC blood-testing, therefore, suggest themselves as feasible and cost-effective diagnostic tools for COVID-19 in low resource settings. In such regions, there is additionally a severe shortage of medical experts to interpret radiological images or blood test results and AI tools provide a way to bridge this gap. In this study we describe the application of AI for analysis of CXR and POC blood-tests in a COVID-19 screening trial, using different AI systems^26,37^. The AI systems were applied to data collected from four sites in low resource settings in Lesotho and South Africa.

## Methods

### Recruitment

The participants included in this work were recruited through two separate trials, TB TRIAGE+ and MistraL as described in more detail below. All data collection was carried out in accordance with relevant guidelines and regulations including the International Council for Harmonisation Good Clinical Practice (South Africa and Lesotho) and the Protection of Public Information Act (South Africa). The study protocols were approved by the Ethikkommission Nordwest- und Zentralschweiz in Switzerland and the National Health Research Ethics Committee of Lesotho (TB TRIAGE+ and MistraL), as well as the Provincial Department of Health of KwaZulu-Natal and the Human Sciences Research Council Research Ethics Committee in South Africa (TB TRIAGE+). All participants provided written informed consent. In case of illiteracy, the adult participant signed with a thumbprint and an independent person signed as a witness.

**Figure 1:**
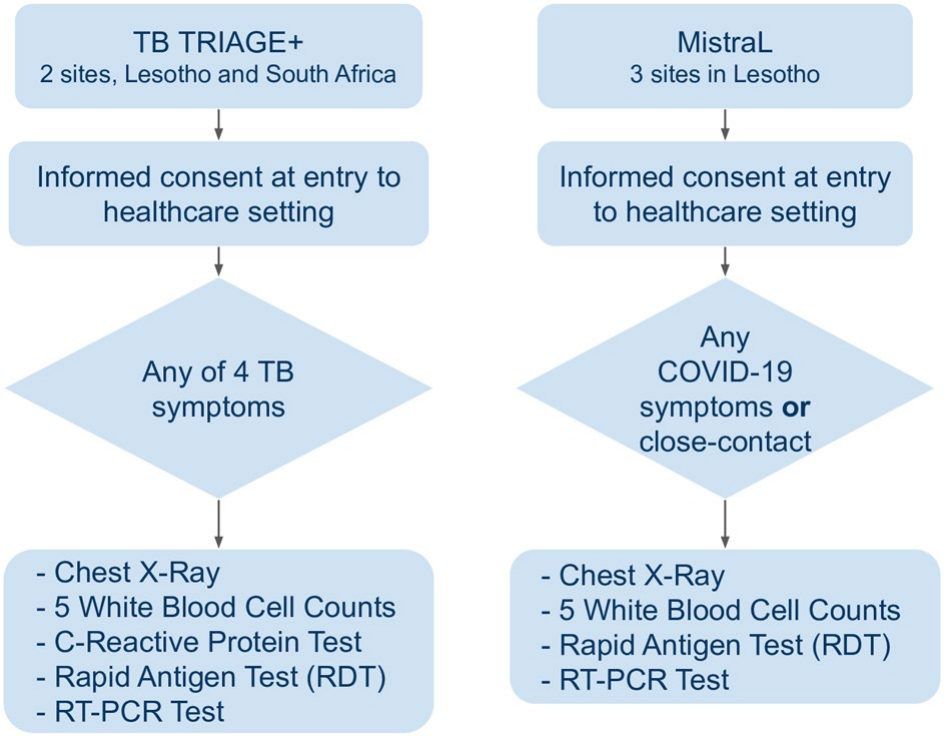
The recruitment process for participants included in this study.

Participants enrolled for COVID-19 screening in either trial had a chest X-Ray acquired as well as a blood test to determine white blood cell (WBC) differential counts (HemoCueR WBC DIFF). This is a point-of-care finger prick blood test which provides counts for the five types of WBC (neutrophils, eosinophils, basophils, monocytes and lymphocytes). All participants received an antigen based SARS-CoV-2 rapid diagnostic test (RDT) (STANDARD Q COVID-19 Ag, SD Biosensor, Republic of Korea) and RT-PCR, both using nasopharyngeal swabs. Participants in TB TRIAGE+ additionally had CRP levels measured in a finger prick blood test (Afinion$^{\textrm{TM}}$ CRP), as part of the standard protocol for that trial. The recruitment process is illustrated in Fig. 1 and described in more detail below. All tests for a single participant were carried out on the same day. Participants under 18 years of age were excluded for the purpose of this study since the AI systems in question had not been validated on paediatric data.

**TB TRIAGE+:** The TB TRIAGE+ trial^43^ was set up to detect tuberculosis (TB) in rural populations in Africa. It is testing the diagnostic accuracy, effectiveness and cost-effectiveness of an AI solution, CAD4TB, for CXR image analysis as well as the CRP blood marker as a means of screening and triaging for TB in community settings. At the height of the coronavirus pandemic, detection of COVID-19 was added to the TB TRIAGE+ protocol since the two diseases have overlapping symptoms and it was expected that both would be present in the community. The participants included in this study were recruited from the Butha-Buthe Government District Hospital, Lesotho and the Caluza Clinic, Pietermaritzburg, KwaZulu-Natal, South Africa if they had any of the four cardinal symptoms of TB (cough, weight loss, night sweats, fever). A CXR of all enrolled participants was acquired as well as a CRP blood test. During the COVID-19 recruitment period, enrolled participants had an additional WBC differential count blood test as well as a SARS-CoV-2 antigen test and an RT-PCR test (both using nasopharyngeal swabs).

**MistraL:** The MistraL trial^44^ was set up specifically in response to the coronavirus pandemic in Lesotho to determine optimal methods of screening for COVID-19. Patients presenting to St Charles Missionary Hospital Seboche or to Government District Hospital of Mokhotlong, as well as initially to the Butha-Buthe Government District Hospital, were screened at the hospital entry point and invited to participate in the MistraL trial if they were experiencing any COVID-19 symptom (fever, cough, fatigue, shortness of breath, sore throat, muscle pain, diarrhoea, loss of taste/smell, weight loss or night sweats) or if they reported close contact with a positive case. A CXR image and a POC WBC differential blood test was obtained for all participants as well as a nasopharyngeal swab rapid antigen test and a nasopharyngeal swab RT-PCR test as the reference standard for COVID-19 infection.

### Artificial intelligence software

The CAD4COVID-XRay AI software^26^ (Delft Imaging, the Netherlands) was developed in April 2020 to detect COVID-19 from chest X-Ray images. This system, which uses deep neural networks for prediction, was first pre-trained using CXR data acquired from various settings prior to the coronavirus pandemic. The system was then fine-tuned (re-trained) for the detection of COVID-19 (defined by RT-PCR results) using CXR images from participants presenting at a Dutch emergency department with respiratory symptoms in March and April 2020. Full details of the training data and procedure can be found in^26^. In testing on a dataset from a different Dutch hospital the system had a performance comparable with six independent radiologists in identifying COVID-19 from the CXR^26^. The CAD4COVID-XRay system takes a single frontal CXR as input and produces a score in the range 0–100 indicating the likelihood of COVID-19. It additionally outputs a heatmap image to illustrate the regions of abnormality on the CXR as determined by the system.

**Figure 2:**
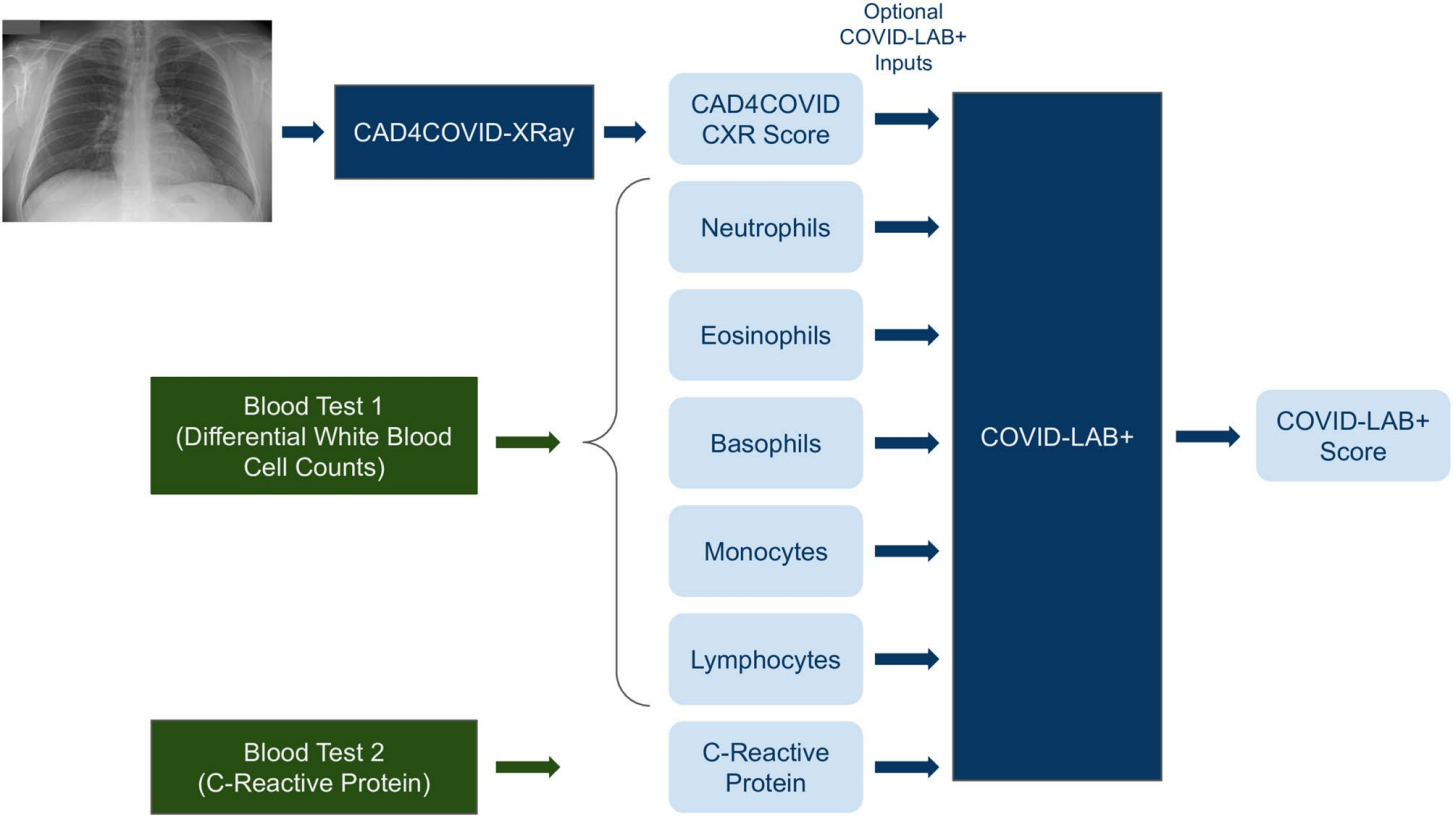
Schematic illustration of the AI systems (dark blue boxes) used in this study. The green boxes indicate a point-of-care blood-test procedure. The pale blue boxes indicate numeric values which are inputs or outputs of the systems as shown. The COVID-LAB+ system does not require all the indicated inputs to be present.

In July of 2020, a second AI system was developed to detect COVID-19 using a combination of data from laboratory testing and CXR^37^. This system uses deep neural networks and is designed and trained to be robust to missing data, and while it is trained on participants with up to 28 inputs (27 laboratory results and a CXR score) it is optimized to provide the best possible results on any subset of these inputs available in a given setting. Training and test data for this system came from the same two independent Dutch hospitals that provided training and test sets for CAD4COVID-XRay. Full details of the network architecture, data and training procedure can be found in^37^. For the purpose of this article we refer to this second AI system as COVID-LAB+ to indicate its purpose to detect COVID-19 using any combination of available laboratory results or CXR score. COVID-LAB+ takes between 1 and 28 numeric values as input—the operator must identify each input as 1 of 27 possible laboratory test results or as a CXR score. It outputs a numeric score (0–100) indicating the likelihood of COVID-19 infection based on analysis of the given inputs. Figure 2 provides a schematic illustration of how these two AI systems operated in our study using the data collected as described in section “Recruitment”. For clarity, this diagram omits mention of other possible inputs to COVID-LAB+, i.e. laboratory tests which were not available in our trials.

Neither CAD4COVID-XRay nor COVID-LAB+ were re-trained for the purpose of this study. There was insufficient data available from our study to do so and there was no evidence to suggest that the systems would not generalise well.

### Radiological reading

To provide a radiological reference for the CXR images in the study a chest radiologist with 8 years of experience was asked to view and rate a subset of the CXR data collected. This subset was chosen to include CXRs from all participants with a positive RT-PCR test result. To balance the data set, each RT-PCR positive participant was then matched with a participant with negative RT-PCR, from the same trial with sex and age matched (within 3 years). In this way the subset of data contained equal numbers of RT-PCR positive and negative participants.

The radiologist was blinded to the RT-PCR results and images were presented in a randomly shuffled order. For each image the radiologist was asked to provide a score from 0 to 3, using the same radiological rating system that was employed in the CAD4COVID-XRay study^26^ (or to rate the image as unreadable). The scores assigned have the following definitions:

0. normal, no finding
1. abnormal but no lung opacity consistent with pneumonia
2. lung opacity consistent with pneumonia (unlikely COVID-19)
3. lung opacity consistent with pneumonia (consistent with COVID-19)

The radiologist performance for COVID-19 prediction is calculated in terms of sensitivity and specificity using different cut-off thresholds on these scores. For this purpose, the scores 0 and 1 are merged, since both represent a clear opinion that no pneumonia related abnormality is present. Since COVID-19 pneumonia is difficult to distinguish from other-cause pneumonia on CXR a cut-off threshold at scores>1 is expected to produce higher sensitivity, whereas with the cut-off at scores>2 specificity is expected to improve.

### AI system analysis

In all cases the RT-PCR result is considered as the reference standard for COVID-19 infection. To determine the most effective screening tools for detection of COVID-19, AI systems using different inputs and combinations thereof are examined as follows: CAD4COVID-XRay: This system requires only a CXR image as input and outputs a likelihood score for COVID-19 (0–100). A receiver operating characteristic (ROC) curve is plotted by applying all possible thresholds to the set of scores, generating sensitivity and specificity points. COVID-LAB+: The COVID-LAB+ system is run using all possible combinations of inputs available. These include:

- CXR+WBC+CRP
- CXR+WBC
- CXR+CRP
- WBC+CRP
- WBC
- CRP

In each case ROC analysis is provided by thresholding on the scores output by the system. Area under the ROC curve is provided for the data from each trial (TB TRIAGE+ and MistraL) separately as well as for the combined datasets. It should be noted that the COVID-LAB+ systems are run on varying datasets, with each dataset being selected to include participants with the required input data parameters available. The sensitivity and specificity of the rapid antigen test used in the MistraL trial is additionally plotted for comparison with the different AI systems.

### Statistical analysis

To make a statistical comparison of the AI system results, de Long’s test^45^ was used. For this comparison, only participants where all test results were available were included, to ensure exactly the same test subjects were provided to every system. Results for TB TRIAGE+ (participants with CXR, WBC, CRP, RT-PCR) and for MistraL (participants with CXR, WBC, RT-PCR) are shown separately. The scores from all tested AI systems were used to determine whether the achieved AUC values are statistically different. The p-values for each comparison are provided and p< 0.05 is considered to represent a statistically significant difference.

## Results

### Recruitment

**Figure 3:**
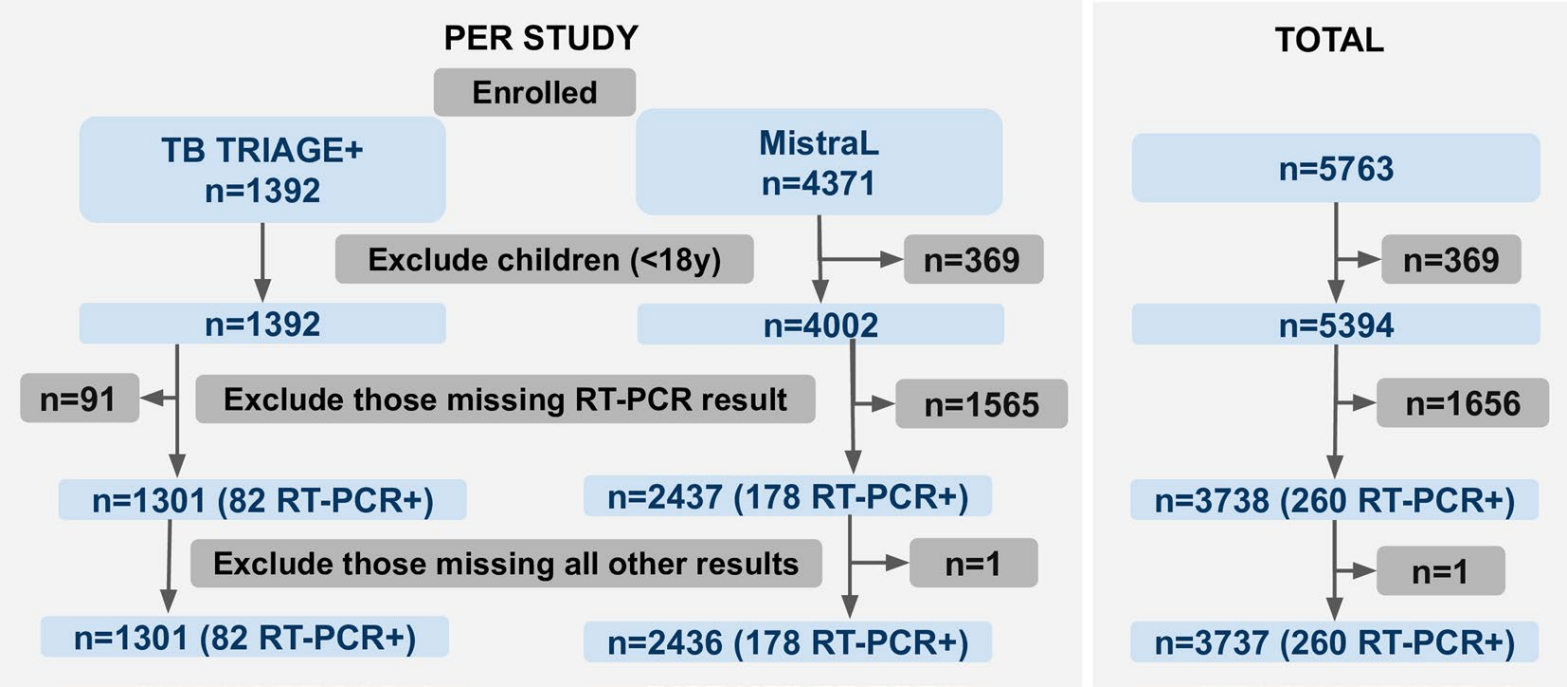
Numbers of participants enrolled, excluded and finally included in our analysis. Figures in brackets indicate the subset which had a positive RT-PCR result.

**Figure 4:**
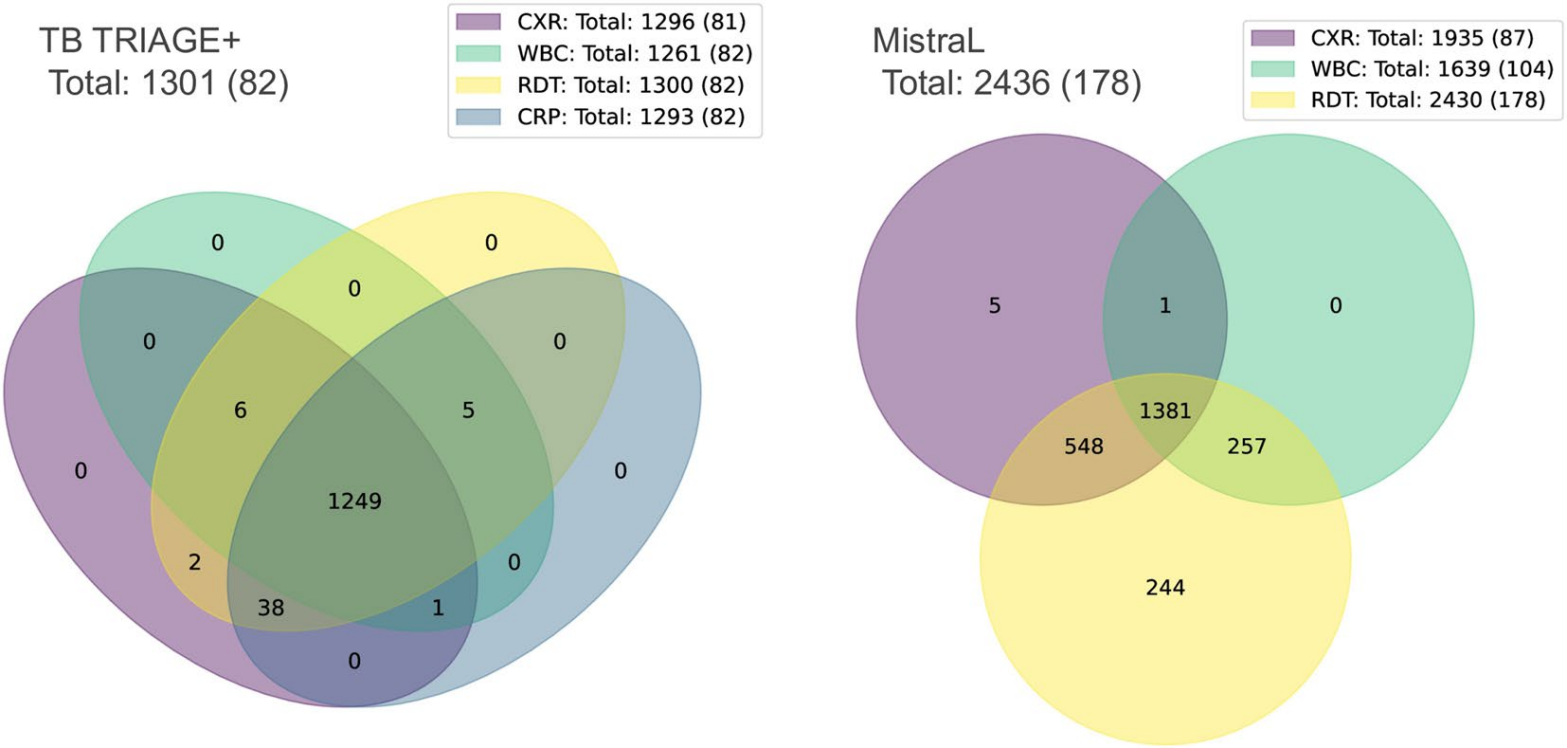
Information on the numbers of participants recruited in each trial and how many results were recorded for the various tests available. Figures in brackets indicate numbers with a positive RT-PCR test. CXR, chest X-Ray; WBC, white blood cell differential count; RDT, rapid diagnostic test (antigen based); CRP, C-reactive protein test.

Data from a total of 5763 participants was collected across both trials between December 2020 and August 2022. Local guidelines in Lesotho were amended to remove the need for PCR testing in late 2021. In keeping with those guidelines, MistraL participants from 19th November 2021 onwards did not routinely receive RT-PCR testing and are thus excluded from this analysis. After the exclusion of children under 18 years of age, 5394 participants remained with RT-PCR results available for 3738 (69%) of these. A total of 260 positive RT-PCR tests were recorded in adults. For pragmatic and operational reasons, some participants did not receive all tests available in the trial in which they were enrolled. Figures 3 and 4 provide detailed information on numbers of participants recruited in each trial and which tests those participants received. One participant with no test results (except RT-PCR) was excluded from further analysis. Table 1 provides additional demographic and clinical information about the participants in the included cohort. Detailed information on the recruitment procedure, and results for the MistraL study can be found in^44^. A publication from the TB TRIAGE+ study is in preparation.

Numbers of participants enrolled, excluded and finally included in our analysis. Figures in brackets indicate the subset which had a positive RT-PCR result.

**Table 1:** Demographic information for the 3737 participants whose data is included in our analysis (as per Fig. 3).

	TB TRIAGE +	MistraL	Total
**Included**	1301 (100%)	2436 (100%)	3737 (100%)
**Gender**			
Female	595 (45.7%)	1475 (60.6%)	2070 (55.4%)
Male	705 (54.2%)	961 (39.4%)	1666 (44.6%)
Ambiguous/Intersex	1 (0.0%)	0 (0.0%)	1 (0.0%)
**Age, median (IQR)**	46 (33–57)	44 (31–61)	45 (32–60)
**BMI, median (IQR)**	22 (19–27)	24 (21–29)	24 (20–28)
**Illness Severity (Self Reporting)**			
Mild / Moderate	1208 (93%)	1772 (77%)	2980 (82.8%)
Severe	91 (7%)	529 (23%)	620 (17.2%)
**Severe Illness (Health Professional Opinion)**			
Yes	11 (0.8%)	37 (1.6%)	48 (1.3%)
No	1290 (99.2%)	2264 (98.4%)	3554 (98.7%)
**Symptoms**			
Cough	109 (8.4%)	852 (39.4%)	961 (27.8%)
Fever	1007 (77.4%)	2016 (99.6%)	3023 (90.9%)
Fatigue	796 (61.2%)	1794 (78.1%)	2590 (72.0%)
Muscle Pain	1120 (86.1%)	2096 (91.2%)	3216 (89.4%)
Loss sense of smell	1180 (90.7%)	1853 (92.2%)	3033 (91.6%)
Other symptoms	1252 (96.2%)	2271 (98.9%)	3523 (97.9%)
**HIV status**			
Negative	653 (50.2%)	1377 (56.5%)	2030 (54.3%)
Positive	628 (48.3%)	540 (22.2%)	1168 (31.3%)
Refused testing	20 (1.5%)	12 (0.5%)	32 (0.9%)
Unknown	0 (0.0%)	507 (20.8%)	507 (13.6%)

### COVID-19 prediction using chest X-Ray

**Figure 5:**
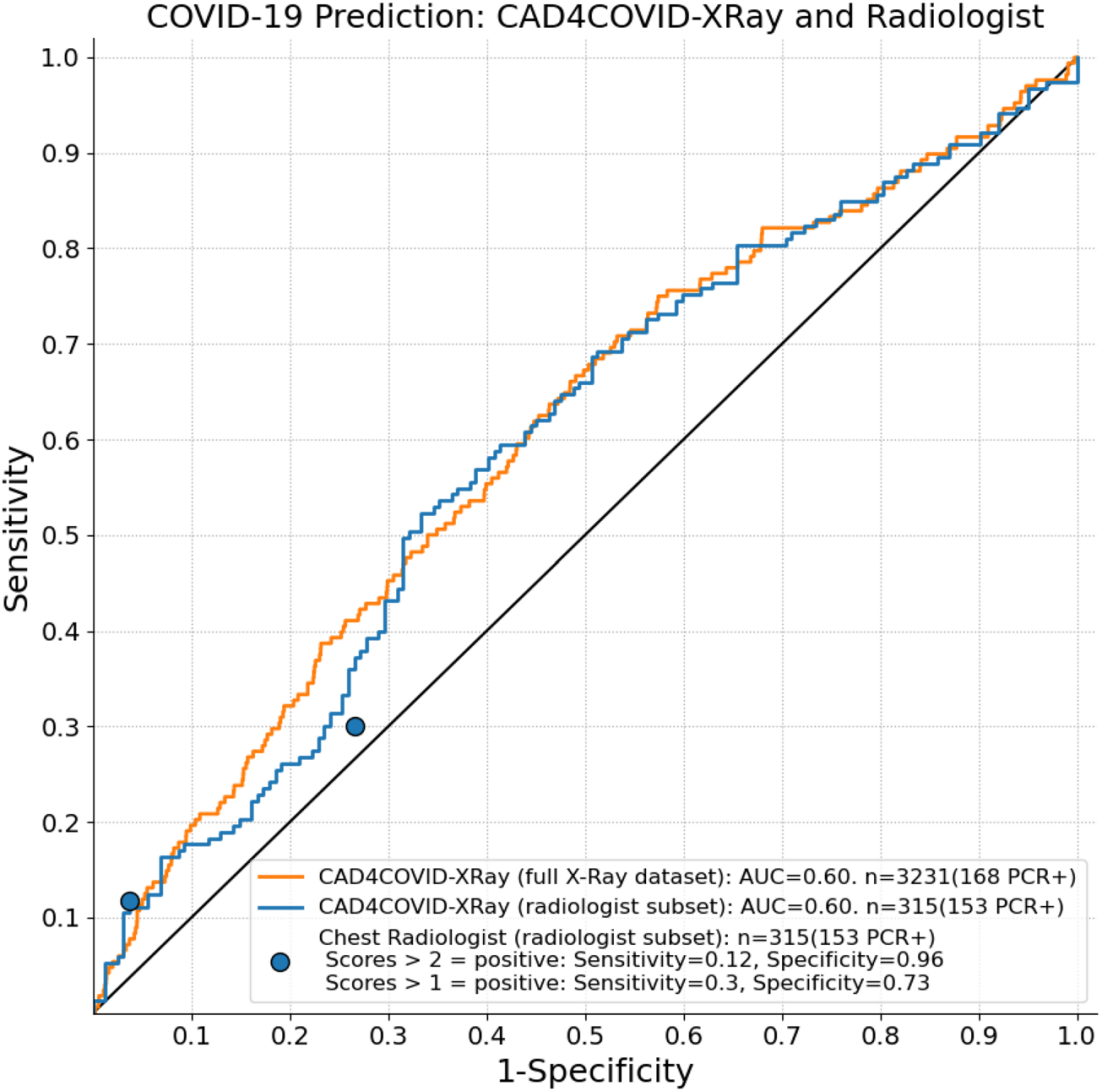
COVID-19 prediction using X-Ray analysis only. The ROC curves show the results for CAD4COVID-XRay using all available CXR images (orange) and a subset selected for radiological reading (blue). The blue points indicate the performance using the scores from the radiologist on this same subset at two cutoff points (i.e. scores>2 considered positive and scores>1 considered positive).

A total of 3231 participants were included with CXR imaging (see Fig. 4), of which 168 had positive RT-PCR tests. A subset of 336 images was selected (including all 168 images from participants with positive RT-PCR) and reviewed by a chest radiologist. Of these, 21 images were deemed unreadable by the radiologist, meaning the image quality was insufficient to draw a conclusion. Table 2 provides a summary of the remaining 315 scores assigned, categorized by RT-PCR results. The chest radiologist obtained a maximum sensitivity of 0.3 with specificity of 0.73 for detection of COVID-19 in this dataset. The CAD4COVID-XRay system achieved an AUC = 0.60 on the same dataset scored by the radiologist, and also AUC = 0.60 when applied to the full dataset of 3231 images. The ROC data is depicted in Fig. 5 along with the sensitivity and specificity achieved by the radiologist.

On the subset of X-ray data from TB TRIAGE+ the CAD4COVID-XRay system achieved an AUC of 0.64, while on that from MistraL the AUC was 0.57 (Table 3).

**Table 2:** Scores assigned by the chest radiologist on the 315 images which he deemed readable. Percentages are calculated per RT-PCR category. For ROC analysis the scores of 0 and 1 are combined since both are interpreted as having no pneumonia-related abnormality.

	RT-PCR positive	RT-PCR negative	Total
Score 0: (normal, no finding)	100 (65%)	97 (60%)	197
Score 1: (abnormal, but non-pneumonia)	7 (5%)	22 (13%)	29
Score 2: (abnormal, possible pneumonia, unlikely COVID-19)	28 (18%)	37 (23%)	65
Score 3: (abnormal, possible pneumonia, likely COVID-19)	18 (12%)	6 (4%)	24
**Total**	**153 (100%)**	**162 (100%)**	**315**

### COVID-19 prediction using COVID-LAB+

**Figure 6:**
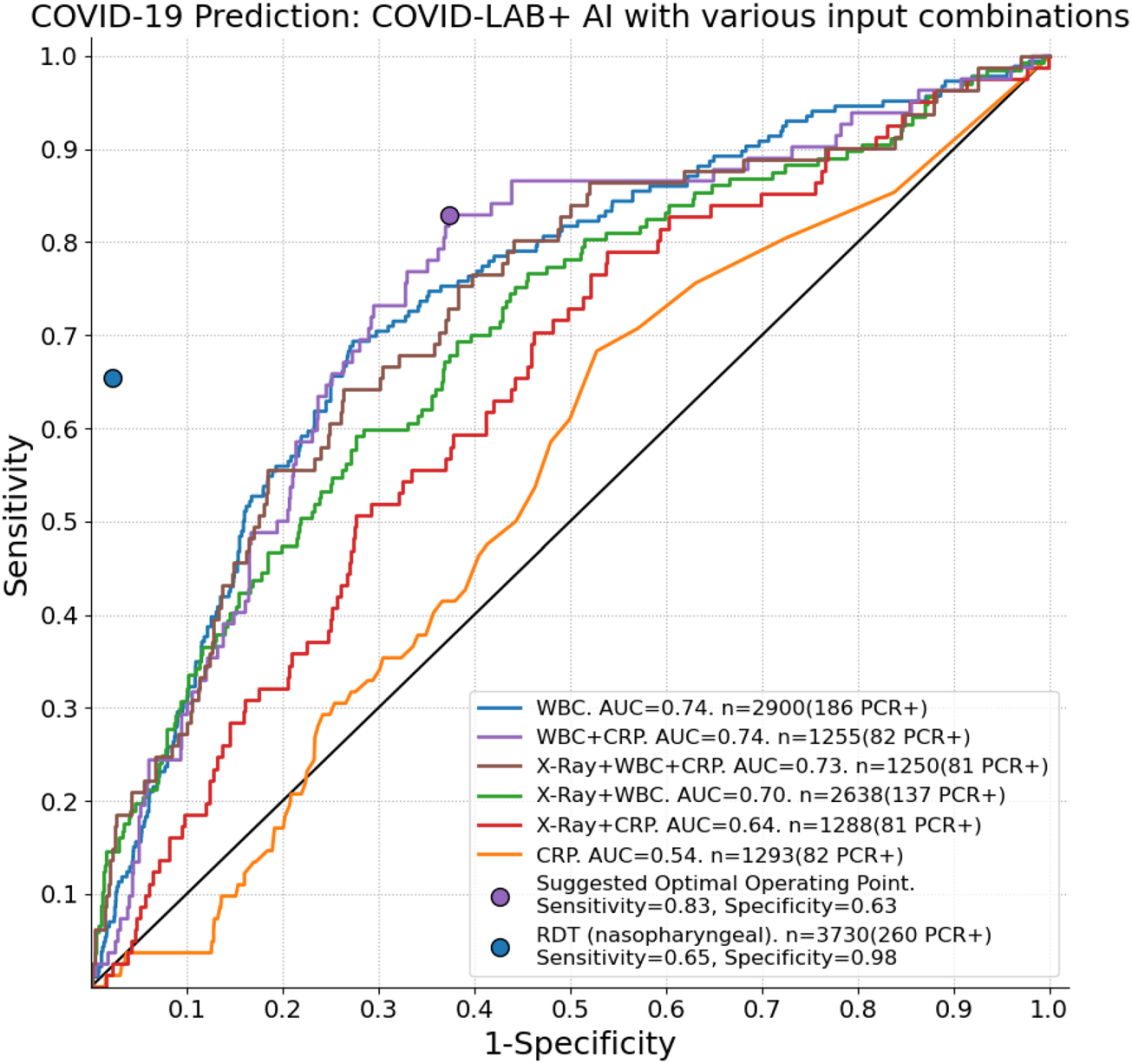
ROC results for the COVID-Lab+ system, run using different combinations of input parameters. Note that different curves are created from different populations, depending on data available for specific input parameters. The sensitivity and specificity obtained by antigen-based testing is additionally plotted. WBC, White blood cell counts; CRP, C-Reactive Protein; RDT, rapid diagnostic test (antigen based).

The COVID-LAB+ AI system was used to predict COVID-19 using all possible combinations of X-Ray and blood tests available. ROC analysis of these experiments is illustrated in Fig. 6, where the sensitivity and specificity of antigen based RDT is also plotted for comparison. At the sensitivity of the RDT (65%), none of the AI systems can achieve a specificity close to that obtained by the RDT (98%). However, the AI systems are capable of operating at higher sensitivity levels, albeit with a reduced specificity. The WBC counts were the most predictive input for a single test, obtaining AUC of 0.74. While CRP alone has a poor performance (AUC = 0.54), when combined with WBC counts, the COVID-LAB+ system could also obtain an AUC of 0.74, boosting sensitivity and specificity at the central part of the ROC curve compared to WBC alone. The suggested optimal cut-off using this system achieved 83% sensitivity and 63% specificity. Only the system with CRP alone (AUC = 0.54) has an improved performance when CXR is added as an additional input (AUC = 0.64).

Comparing results on the subsets of data from TB TRIAGE+ and MistraL respectively (Table 3) the systems using X-Ray perform similarly in both, with a slightly higher AUC on the TB TRIAGE+ dataset, while the system using WBC alone has an identical AUC of 0.74 on both datasets.

**Table 3:** AUC scores for all systems, per individual study (TB TRIAGE+ and MistraL) and combined. Note that CRP was not collected in the MistraL study, resulting in some empty cells. ROC curves for combined datasets are provided in Figs. 5 and  6.

	System performance (AUC) by dataset		
	TB TRIAGE+	MistraL	Combined
CAD4COVID-XRay	0.64 (n = 1296 (81 PCR+))	0.57 (n = 1935 (87 PCR+))	0.60 (n = 3231 (168 PCR+))
WBC	0.74 (n = 1261 (82 PCR+))	0.74 (n = 1639 (104 PCR+))	0.74 (n = 2900 (186 PCR+))
WBC+CRP	0.74 (n = 1255 (82 PCR+))		0.74 (n = 1255 (82 PCR+))
X-Ray+WBC+CRP	0.73 (n = 1250 (81 PCR+))		0.73 (n = 1250 (81 PCR+))
X-Ray+WBC	0.73 (n = 1256 (81 PCR+))	0.65 (n = 1382 (56 PCR+))	0.70 (n = 2638 (137 PCR+))
X-Ray+CRP	0.64 (n = 1288 (81 PCR+))		0.64 (n = 1288 (81 PCR+))
CRP	0.54 (n = 1293 (82 PCR+))		0.54 (n = 1293 (82 PCR+))

### Statistical analysis

The set of 1250 participants with all test results needed for AI evaluation (RT-PCR, CXR, WBC, CRP) was included for statistical testing. Scores for all AI systems on this subset of participants were used to generate AUC values and determine whether the differences in these AUC values were statistically significant^45^. Table 4 indicates the AUC values on this dataset, which were very similar to those achieved on the full datasets. This table also provides p-values for each system comparison. All other AI systems are significantly better than the system using CRP alone as a predictor. Furthermore, any system including WBC as an input is significantly better than any system that does not include WBC. No statistically significant difference could be detected between the four systems which include WBC as an input (which also have the highest AUC values among all those tested.)

**Table 4:** Results of statistical comparison of the scores from different systems on data from TB TRIAGE+ and MistraL respectively. Values shown are p-values from deLong^45^ testing. Comparison was performed on (a) results from the 1250 TB TRIAGE+ participants with CXR, WBC, CRP and RT-PCR test results available (81 PCR+) and (b) results from the 1382 MistraL participants with CXR, WBC and RT-PCR test results available (56 PCR+) (shown in *[italics]* throughout). AUC values using only these specific participants are indicated in the table (using *[italics]* for MistraL data). The * symbol is used to indicate p-values lower than 0.05 (statistically significant difference between the systems)..

		COVID-LAB+					
		WBC (AUC = 0.74 * [0.69]*)	WBC+ CRP (AUC = 0.74)	X-Ray+ WBC+ CRP (AUC = 0.73)	X-Ray+ WBC (AUC=0.73 * [0.65]*)	X-Ray+ CRP (AUC = 0.64)	CRP (AUC =0.55)
	CAD4COVID-XRay (AUC = 0.65 * [0.55]*)	0.02* [0.01]*	0.01*	1.65E−04*	1.41E−04* [1.65E−03]*	0.10	0.01*
COVID-LAB+	WBC (AUC = 0.74 * [0.69]*)		0.79	0.63	0.74*[0.16]*	0.01*	1.66E−05*
	WBC+CRP (AUC = 0.74)			0.49	0.62	4.04E−03	8.37E−07
	X-Ray+WBC+CRP (AUC = 0.73)				0.33	4.81E−05	4.33E−06
	X-Ray+WBC (AUC = 0.73 * [0.65]*)					6.52E−05	9.50E−06
	X-Ray+CRP (AUC = 0.64)						0.01*

## Discussion

In this work, AI systems for detection of COVID-19 have been applied to input data collected from participants in resource-constrained settings in Africa during the COVID-19 pandemic. Although many studies have been published on the use of AI systems for COVID-19 detection, to our knowledge this is the first published application of AI for COVID-19 screening in an African setting, and one of few applied outside a hospital setting to participants without severe respiratory symptoms at presentation.

At the time of commencing data collection (December 2020) there was an expectation that, during the study, waves of COVID-19 would be encountered, where case numbers would be high and disease would often be severe. In this context, a much higher incidence of radiologically evident COVID-19 pneumonia was expected, suggesting AI for chest X-ray (CAD4COVID-XRay) as a suitable predictor of infection. This was the scenario already encountered in many countries around the world, and in which CAD4COVID-XRay had been demonstrated to perform well^26^. In fact, there was an overall low incidence of COVID-19 cases (7%) during the study and the majority of these had mild symptoms. This may be attributed to fluctuations in variants circulating and in population immunity which substantially affect disease prevalence and severity. For this reason, CAD4COVID-XRay had a relatively poor performance at identifying RT-PCR+ participants, since they rarely had pneumonia visible in the lungs. This is supported by the radiological reading where the chest radiologist found that 100 of 153 images from RT-PCR+ particpants were completely normal in appearance, and only 18 of the 153 were suggestive of COVID-19 pneumonia. The similarity in performance between CAD4COVID-XRay and the radiologist suggests that the AI system was operating correctly (did not require re-training) but that the information pertaining to COVID-19 (signs of pneumonia) was simply absent from the majority of X-ray images. While many studies have suggested that chest X-ray analysis can identify COVID-19 infection (both with or without AI)^1,5,23–34^ it is important to clarify that this is only applicable in settings where disease is severe and participants report with acute respiratory symptoms indicative of pneumonia which would result in relevant radiological findings.

The COVID-LAB+ system was employed to investigate the potential of combinations of POC tests for prediction of COVID-19. In designing the study, differential WBC was selected as an inexpensive POC blood test which showed promise as a COVID-19 predictor in earlier studies^9,37^. CRP was additionally included in this work since it was collected as part of the TB TRIAGE+ protocol and had also been indicated as a potentially useful marker of COVID-19 prognosis^46–48^. Our results indicate that WBC count alone is a strong predictor of COVID-19 infection and all AI systems using it performed significantly better than those without it. No significant difference was found between any of the four systems that incorporated WBC (alone or in combination) although the suggested optimal operating point (sensitivity = 0.83, specificity = 0.63) was achieved by combining CRP with WBC.

The AUC value (0.73) achieved by COVID-LAB+ using the combination of X-Ray + WBC + CRP is substantially lower than that reported using an identical set of input variables in the first publication on the system (AUC = 0.919)^37^. This is likely related to the fact that the data in that study (both training and test data) was collected at emergency departments in Dutch hospitals at the beginning of the pandemic, where patients presented with severe respiratory complaints. The variants in circulation at that time, as well as lack of population immunity typically resulted in more severe disease manifestation. Further investigation is required to verify whether severity of illness was the only reason for the disparity in AUC values between this study and the earlier one.

At the time that this study commenced, antigen-based testing was not commonly used or well validated, although it has since become commonplace in many parts of the world. The performance of antigen RDTs in this study (sensitivity = 0.65, specificity = 0.98) concurs with many previous works which report relatively low sensitivity values but very high specificity^49^, although there is evidence that antigen based tests are more effective in subjects with high viral load^50^. In many environments, however, such as healthcare settings, a test with higher sensitivity is desirable, even for cases with milder or earlier stage disease, in order to reduce the possibility of COVID-19 transmission among vulnerable populations. A triage test with high sensitivity could enable accurate selection of participants recommended to receive RT-PCR testing, for example. Future investigation should determine whether antigen-based RDT results could be usefully incorporated as an input to our COVID-LAB+ system, potentially improving specificity without loss of sensitivity. Although the need for tests which can detect SARS-CoV-2 has reduced over the course of time, the benefits of combining different types of inputs (such as blood markers and radiological findings) could also be investigated in other applications such as the accurate detection of TB, for example.

Our study had some limitations. Since the data was collected over a period of almost 2 years there were fluctuations in the prevalence of COVID-19, population immunity, and in the circulating variants during that period^44^. No data on participant vaccination status was collected although COVID-19 vaccines were introduced in South Africa and Lesotho on a phased basis starting in 2021. Variations in such factors may have affected the predictive values of the diagnostic tests used^51^. Furthermore, a substantial number of those recruited in the MistraL study (39%) had no result for RT-PCR as they were recruited after November 2021 when local guidelines indicated antigen-based RDT instead of RT-PCR testing. This may result in imbalances in the data included from the two trials, in terms of circulating variants and prevalence. Finally, due to limited numbers of positive COVID-19 cases and limited scope of this manuscript, we did not perform subgroup analysis using age, HIV-status, clinical severity or other factors. Future work may indicate the role of these factors in diagnosis.

Diagnostic AI systems in resource-constrained settings are limited, compared to those used elsewhere, in that the input data must generally be acquired at low-cost, at point-of-care and by personnel with minimal training. A review by Hirner et al.^52^ of tools for COVID-19 screening, triage and severity scoring found none that had been validated in low or middle income countries, despite 51 of the identified tools being considered feasible for use in such settings. This study is, to the best of our knowledge, the first to demonstrate that such tools can be successfully deployed in a low-resource setting. Participants were people presenting at local medical facilities and all tests were carried out on the same day, using temporary facilities on-site, without interruption to their standard care. This demonstrates the feasibility of collecting such point-of-care imaging and blood tests in low resource settings where patient follow-up is difficult and therefore cost-effective screening tools with immediate results are of utmost importance. This novel study lays the foundations for future AI development for low resource settings, with the potential, in the future, to detect a variety of conditions based on low-cost point-of-care testing.

## Data Availability

A spreadsheet containing all data used for the analysis shown in this work will be placed in a permanent public repository on zenodo.org upon publication. All data will be fully anonymized. Details of the repository, including DOI will be provided here at that time.
